# Seasonal Variation of Malaria Parasite Density in Paediatric Population of North Eastern Nigeria

**DOI:** 10.5539/gjhs.v4n2p103

**Published:** 2012-03-01

**Authors:** L. M. Samdi, J. A. Ajayi, S. Oguche, A. Ayanlade

**Affiliations:** Unit of Parasitology and Entomology Department of Zoology, University of Jos, P.M.B 2084 Jos, Plateau State, Nigeria E-mail: samdilaz@yahoo.com; Unit of Parasitology and Entomology Department of Zoology, University of Jos, P.M.B 2084 Jos, Plateau State, Nigeria E-mail: ajayi@unijos.edu.ng; Univ. of Jos Teaching Hospital, Univ. of Maiduguri Teaching Hospital Maiduguri Borno State, Nigeria E-mail: soguche2001@yahoo.com; Department of Geography Obafemi Awolowo University, Ile-Ife, Nigeria E-mail: sinaayanlade@yahoo.co.uk

**Keywords:** Malaria, Northeastern Nigeria, *Plasmodium falciparum*, Children, *Anopheles gambiae*

## Abstract

Malaria is a major cause of morbidity and mortality in children and adults in the Sudano Sahel of Northeastern Nigeria with the highest prevalence of malaria in pregnancy of 64.5 in Nigeria recorded in this region. This study was carried out in 2003 and 2004 to provide parasitological baseline data for the development of Malaria Early Warning System (MEWS) for the surveillance of type I epidemic caused by meteorological conditions and to provide data for timing malaria key vector control measures such as Indoor Residual Spraying (IRS) for maximum effect. Clinical information about malaria cases were used in this study. In all 692 children aged 6 to 96 months were screened for *Plasmodium* infection and used for the analysis. The results showed that the majority of infected children (68.06%) were aged 12-60 months and their asexual parasite density (ap/ u1) was between 100-500 ap/u1 of the whole blood. The month of September recorded the highest Geometric Mean Asexual-Densities (GMPD) of 13,655 while the lowest parasite densities were observed at the peak of the dry season, especially during the months of March and April. Significance difference (p<0.05) was observed between the sexes in infection rate. It is obvious that male children have higher infection rate (about 67.5%), than while female children (32.5%) regardless of climate seasonality. Designing a malaria early warning system and providing baseline parasitological data for timing of spraying cycles for key malaria vector control measures such as Indoor Residual Spraying (IRS) should be encouraged to complement other effective malaria control strategies. Hence the need for this investigation.

## 1. Introduction

Malaria currently accounts for nearly 110 million clinically diagnosed cases per year in Nigeria and responsible for about 60% of outpatient visits; 30% of hospital admissions. Mortality due to malaria in children is estimated at 300,000 children each year; up to 11 % of all maternal mortality is due to malaria (FMOH, 2009).

Malaria is hyper endemic throughout the semi-arid zone of Borno State and other states of the Sahel in Nigeria. Peak transmission occurs during the middle to late rainy season (Molta *et al.*, 1995). Studies conducted in Maiduguri town indicated a mean prevalence of 35.2% and 27.26% respectively (Molta *et al.*, 1993; Samdi *et al.*, 2005). Generally, in areas of low endemicity, the incidence of infection is far more sensitive to climate changes (Martens *et al.*, 1995). Though the possible *Anopheles* mosquitoes’ vector of the northeast include *A. gambiae, A. funestus, A. pharoensis. A. squamosis, A. coustani and A. ziemanni* (Pull and Gramiccia, 1976; Gadzama, 1983). Polymerase Chain Reaction (PCR) and ELISA recently showed that *An. arabiensis* is the predominant member of the *Anopheles gambiae* complex and the major vector of malaria in north-eastern Nigeria (Samdi *et al.*, 2006). However, detailed studies of the interaction of rainfall and malaria in the general community in Africa are limited. Studies on the interaction between climate and malaria have focused in recent years, on the potential role of climate change in determining recent increases in malaria transmission in highland areas in Africa where temperature rather than rainfall has been the parameter of greatest interest (Ayanlade *et al.*, 2010). In north-eastern part of Nigeria several studies have shown that monthly figures of malaria among in-patients show seasonal fluctuations; low values characteristic of the dry season and high values in the rainy season (Pull and Gramiccia, 1976; Molta *et al.*, 1995; Oguche *et al.*, 2010). This is not surprising as the biting density and infectivity of the vector intensify during the rainy season. Accordingly, malaria related morbidity and mortality show seasonal trends with peaks in the wet season and a low level in the dry season (Gadzama, 1983; Molta *et al.*, 1995; Oguche *et al.*, 2006).

This study aims to determine malaria parasitaemia in children under the age of 8 years during the two major seasons (dry and rainy seasons) of the study area and also determine the relationship between malaria parasitaemia and seasons. This is to guide preventive, management and key malaria vector control strategies such as Indoor Residual Spraying (IRS) whose spraying cycle is determined by malaria seasonality and climatic conditions and other malaria control strategies such as malaria Early Warning System (MEWS) for this part of Nigeria.

## 2. Materials and Methods

### 2.1 Study Area

This study was conducted at the Emergency Paediatric Unit (EPU) of the University of Maiduguri Teaching Hospital (UMTH), Maiduguri, Nigeria. The UMTH serves as a referral centre catering for the needs of the entire northeast. Maiduguri is located on latitude 11^0^ 40’ N and longitude 13^0^ 05’ E, and altitude of 305 m with mean annual rainfall of 650 mm. The town is inhabited by about 877,925 people. Although this area lies 4 in the sudano-Sahelian zone and experiences a marked seasonal variation in transmission of malaria, malaria occurs all year round, with peaks during the middle to late rainy season (BOMOH 1988; Molta *et al.*, 1996; Oguche *et al.*, 2001). It has a short rainy season lasting from July to September /October and a long dry season from October/November to May/June. The dry season has two periods to it; a cold harmattan period from November/December –January and the hot period, February/March- April/May (Molta *et al.*, 1995).

Both climatic and clinical data over a period of one year were used in this study. Standard meteorological data recorded by the Nigeria Meteorological Agency (NIMET), Federal Ministry of Aviation Weather Station Maiduguri International Airport was used. Daily maximum and minimum temperatures, relative humidity and rainfall taken covering the period of the study (18^th^ June 2003 to 18 June 2004) were used. Malaria data were collected from the Emergency Paediatric Unit (EPU) of the University of Maiduguri Teaching Hospital (UMTH), Maiduguri, Nigeria In all, 692 infants and children under 8 year old (6-96 months) that were present at the EPU were consecutively and routinely screened. The World Health Orsganization (WHO, 1996; 2003) procedure was adopted for the detection of *Plasmodium* positive cases and identification of malaria parasites. Chi-square (X^2^) test analysis was carried out to compare proportions of relationship between climate parameter and malaria incidence. Seasonal correlation between malaria parasitaemia in routinely screened for plasmodium infection with rainfall variability were carried out. Moreso, Giemsa stained thick blood films were used for parasitological assessment and parasite densities were estimated using an average white blood cell count of 8000 per ul (WHO, 1996). The parasitaemia per ul was calculated by using the WHO (1996) formula:


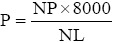


Where P represents Parasitaemia (per ul), NP is the number of Parasites and NL is the number of Leukocytes Chi χ^2^ test was carried out to compare proportions, paired sample t –test (with level of significance at 0.05 was used to compare differences between the seasons. Pearsons correlation was used to indicate the relationship between malaria parasitaemia and the two major seasons.

### 3. Result

Table 1 shows the *Plasmodium falciparum* (Asexual Parasites) infection in relation to the sex of the Children while Table 2 presents the Malaria Parasite Density in relation to the age of the children. The results show that out of a total of 692 severely-ill (malaria cases) children consecutively over a full year, 169 children were positive for malaria parasites. Observation also shows that malaria occurrence differs by sexes- male or female. Out of 397 male children malaria record observed, 114 were positive while only 283 were negative. For female children out of 295 cases, 55 were positive while 240 were negative. The proportions of male that are positive are higher than that of female. Significance difference (p<0.05) was also observed between the sexes with male children having higher infection rate of about 67.46%, while females have about 32.54% (Table 1). It is also obvious from this result that the levels of parasitaemia (asexual parasite density, ap/ul) were significantly related to age (p<0.05). It is evident from Table 2 that the majority of infected children (68.60%) were aged between 12-60 months and their asexual parasite densities were between 1000-5000 ap/ul of whole blood.

**Table 1:** *Plasmodium falciparum* (Asexual Parasites) infection in relation to sex of children

Sex	Positive		Negative		Total	
	No	%	No	%	No	%
Male	114	67.5	283	54.1	397	57.4
Female	55	32.5	240	45.9	295	42.6
Total	169	100.0	523	100.0	692	100.0

χ^2^=2.96 df=1 p<0.05 (Significant)

**Table:** Table 2. Malaria Parasite Density in relation to age in children

Age (months)	Frequency of patients in density classes								
	<1000	1000-5000	>5000	Positive Freq	%	NegativeFreq	%	Total Freq	%
6-11	19	3	0	22	13.01	20	3.82	42	6.07
12-60	116	7	6	129	76.33	342	65.39	471	68.06
61-96	18	0	0	18	10.65	161	30.78	179	25.87
Total	153	10	6	169	100	523	100	692	100

χ^2^ = 7.34 df=2 P<0.05

Tables 3, 4 and 5 present the relationship between climate seasonality and variation in parasitological variation in children. It is obvious that there is significant relationship between climate and malaria occurrences. The results also shows highest geometric mean asexual parasite densities (GMPD) during the rainy season (Table 3) while low mean asexual parasite densities were observed during the peak of the dry season in the study area (Tables 4 and 5). The study area experiences highest rainfall during the month of September. At this peak of rainfall season, the results show highest GMPD record. For example, it is obvious from Table 3 that the month of September recorded the highest GMPD of 13,655 which is the highest proportion. The results also show that lower GMPD were recorded during the peak of dry season when there was little or no rainfall. In the study area, months of March, April and May are periods of little rainfall. Moreover, it is obvious from the result (Table 5) that the period of low GMPD were observed at the peak of the dry season corresponding to the months of March, April and May with lower densities of 0.98, 7.70 and 4.69 respectively.

**Table 3:** Parasitological data on asexual stages of malaria parasites in 692 children of ages 1-8 years of the Emergency Pediatric Unit University of Maiduguri Teaching Hospital in the months during the rainy season

Season	Months	No Examined.	No. Positive for malaria parasites (%)	No negative for malaria parasite (%)	Geometric mean asexual Parasite Density Asexual (GMPD) parasite/ul	Parasite Density range (Asexual parasite/ul)	Frequency of patients in density classes Asexual parasite/ul		
							<1000	1000-5000	>5000
Rainy Season	June	30	16(53.3)	14 (46.7)	64	16-400	16	0	0
	July	19	9 (47.4)	10 (52.6)	107	16-1,613	8	1	0
	Aug.	68	14 (20.6)	54 (79.4)	353	32-12,280	9	4	1
	Sept	36	10 (27.8)	26 (72.2)	13,655	16-111,111	5	2	3
	Total	153	49 (32.1)	104 (67.9)			38	7	4

**Table 4:** Parasitological data on asexual stages of malaria parasites in 692 children of ages 1-8 years of the Emergency Pediatric Unit University of Maiduguri Teaching Hospital during the cold dry season

Season	Months	No. Examined	No. Positive for malaria parasites (%)	No. negative for malaria parasite	Geometric Mean asexual Parasite Density (GMPD) per/ul	Parasite density range (per/ul)	Frequency of patients in density classes (Asexual parasite/ul)		
							<1000	1000-5000	>5000
Cold Dry Season	Oct	55	14(25.5)	41(74.5)	4916	16-250,000	11	0	3
	Nov	62	10 (83.9)	52 (83.9)	1,695	48-102,564	9	0	1
	Dec	89	32(64.1)	57(64.2)	655	16-1,600	31	1	0
	Jan	52	7 (13.5)	45(86.5)	2,720	16-176,000	6	0	1
Total	4 months	258	63 (24.4)	195(75.6)			57	1	5

**Table 5:** Parasitological data on asexual stages of malaria parasites in 692 severely-ill children of ages 1-8 years at the Emergency Paediatric Unit, UMTH, Maiduguri, Nigeria during the hot warm season

Season	Months	No. Examined	No. Positive for malaria parasites (%)	No. Negative for malaria parasite (%)	Geometric mean asexual parasite density/(Asexual parasite/μl)	Parasite density range (Asexual parasite/μl)	Frequency of patients in density classes (Asexual parasite/μl)		
							<1000	1000-5000	>5000
Hot/warm season	Feb	90	18 (20.0)	73 (80.0)	28.98	16 – 1600	18	0	0
	Mar	65	4 (6.5)	61 (93.85	0.98	0 – 64	4	0	0
	Apr	29	8 (38.0)	21 (62.0)	7.17	16 – 48	8	0	0
	May	58	9 (15.5)	49 (84.6)	4.69	16 – 94	9	0	0
	Jun	49	16 (32.7)	33 (67.3)	16.98	16 – 400	16	0	0
	Total	281	54 (19.3)	227 (80.7)			54	0	0

Paired sample t-test (with a level of significance at 0.05) showed a significant difference between the seasons (P=6.82), Pearsons coefficient of correlation showed a weak relationship between malaria parasitaemia and the seasons.

## 4. Discussion

It is important to understand the seasonality of malaria parasitaemia in order to prepare for and implement integrated malaria control measures. This preparation should be well ahead of the peak malaria transmission periods targeting the mosquito and the infected people simultaneously since malaria transmission depends principally on both. The outcome of this study agrees with the results of the earlier studies by Thomson *et al*. (2005) and Ayanlade *et al*. (2010) that the seasonality of climate greatly influences the seasonality of malaria transmission. This study has further confirmed that malaria parasitaemia in the Sahel varies with a clear seasonal pattern in climate. This is in agreement with Pull and Gramiccia (1976) and Molta *et al.*, (1995) that the relatively dry northern savanna of the country demonstrates strong seasonality in malaria transmission. Therefore, the north has unstable (hypo endemic or mesoendimic) malaria. Oguche (2001) further demonstrated this strong seasonality in a study with cerebral malaria of the pattern of childhood cerebral malaria in northeastern Nigeria. Ninety- five percent of the patients presented between June and November had malaria with a peak in October.

It has been reported in the previous studies that seasonal fluctuations in rainfall affects the occurrence of malaria in some parts of Africa. For example, Molta (1995) observed that the monthly figures of malaria among in-patients in the Sahel show seasonal fluctuations and that low values are characteristic of the dry season and high values are of the rainy season. This is in contrast with the wet forest areas of Nigeria where malaria transmission occurs at high level all year round (FMOH, 1991). Though, Enosolease and Awodu (2003) have also reported that malaria parasitaemia fluctuates throughout the year without any clear pattern and devoid of seasonality in Benin, Southern Nigeria. The distribution of malaria amongst various age groups agrees with reports by White and Ho (1992) and FMOH (1991), that young children aged 0-4 years support the highest asexual and sexual parasite densities. Thus, infants suffer a disproportionately high rate of infection while older children are at lower risk, presumably for immunological reasons.

### 4.1 Limitation

This work was limited by the inability to obtain detailed and quality satellite meteorological data to correlate with the results of malaria parasitaemia though some manually compiled meteorological data was obtained from the local weather station it was not as useful as expected.

## 5. Conclusion

The relationship between climate parameter and malaria occurrences were examined in this study. This study showed that the highest geometric mean parasite density was observed in September. The month of September is known as a period of sudden mosquito population outburst due to frequent rainfall with fairly long periods of sunshine which increase the opportunity for mosquitoes’ prolific breeding. The findings presented in this study agree with the results of previous studies that the vector densities, in some parts of northeast region are associated with seasonal changes in rainfall. Findings of this study are valuable contributions to an already existing pool of baseline data. It could guide in designing of control programmes that are locally adapted and technically/financially feasible such as Malaria Early Warnings Systems (MEWS) which is extremely relevant where malaria transmission is unstable. Indicators such as temperature, humidity and rainfall, for this purpose is now receiving renewed attention (WHO, 2001) and other key malaria vector control strategies currently being scaled up by the Federal Ministry of Health such as Indoor Residual Spraying (IRS) whose timing of spray cycles depend on climatic indices like rainfall patterns too. Other intervention strategies such as insecticide impregnated bed nets are currently advocated by the Roll Back Malaria (RBM) initiative, behavioural changes, environmental management and larviciding (Curtis, 1991) could be designed based on these data.

**Figure 1:**
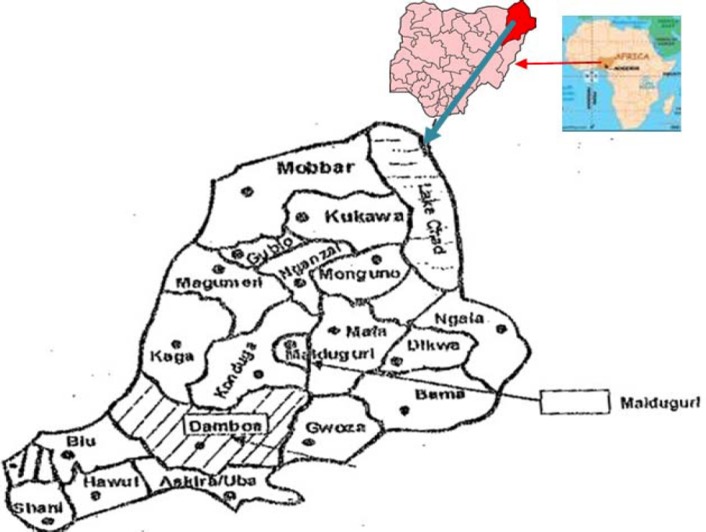
Map of Borno state of Nigeria showing study location
